# Neurodevelopmental disorders in children seeking obesity treatment- associations with intellectual ability and psychiatric conditions

**DOI:** 10.3389/fpsyt.2024.1332598

**Published:** 2024-08-19

**Authors:** Maria Dellenmark-Blom, Kajsa Järvholm, Lovisa Sjögren, Anna Levinsson, Jovanna Dahlgren

**Affiliations:** ^1^ Department of Pediatrics, Institute of Clinical Sciences, Sahlgrenska Academy, University of Gothenburg, Gothenburg, Sweden; ^2^ Department of Psychology, Lund University, Lund, Sweden; ^3^ Childhood Obesity Unit, Skåne University Hospital, Malmö, Sweden; ^4^ Department of Pediatrics, Hallands Hospital Halmstad, Halmstad, Sweden; ^5^ School of Public Health and Community Medicine, Institute of Medicine, Sahlgrenska Academy, Gothenburg University, Gothenburg, Sweden; ^6^ Centre de Recherche du Centre Hospitalier de l’Université de Montréal, Montreal, QC, Canada; ^7^ Region Västra Götaland, Regional Obesity Center, Gothenburg, Sweden

**Keywords:** obesity, neurodevelopmental disorders, intellectual ability, cognition, depression, anxiety, psychiatric disorders

## Abstract

**Background:**

Neurodevelopmental disorders (NDD), psychiatric comorbidity and cognitive deficits are commonly seen in children with obesity; however, little is known about the overlap between these conditions. This study aimed to examine the undiagnosed and diagnosed frequency of NDDs and explore its association with psychiatric conditions and general intellectual ability (IQ) in children presenting for obesity treatment.

**Methods:**

In this observational study at two outpatient obesity clinics during 2018-2019, 80 children (8-17 years) were consecutively recruited, and screened for NDD unless already diagnosed with an NDD. A psychiatric unit evaluated children who screened positive for NDD. Diagnoses and clinical background factors were collected from medical records. IQ was assessed with the Weschler Intelligence Scales and internalizing symptoms were assessed using the Beck Youth Inventories. Associations between background factors, IQ and internalizing symptoms were explored in relation to having an NDD or not.

**Results:**

We found that 47/80 children had at least one NDD. Children with a diagnosed NDD before study start (n = 30) had significantly more comorbidities than children diagnosed after the study screening (n = 17) (*P* = .01). Greater cognitive impairment was seen in children with NDD compared with children without an NDD (*P* = .01). Also, 33/73 participants self-reported substantial internalizing symptoms. At follow-up, 21/79 participants, in addition to NDD, had been diagnosed with another psychiatric disorder. Ten of these were children that had been diagnosed with an NDD before study start.

**Conclusion:**

The overlap between NDD, cognitive deficits and psychiatric conditions in children with obesity is an important consideration for clinical practice. Screening for these conditions may be necessary when providing targeted interventions.

## Introduction

Pediatric obesity is a complex multifactorial disease that is considered a global challenge due to its high prevalence and long-term risk of adverse health outcomes. Early identification and multicomponent behavioral interventions are imperative (1, 2), but high attrition (40-80%) is a common problem in obesity treatment (2, 3).

Most multicomponent behavioral interventions require the child and the family to integrate new strategies in everyday life to balance energy intake against energy expenditure. Successful establishment of such strategies may be dependent on the patient’s behavioral and cognitive abilities (1). However, a substantial proportion of children presenting for obesity treatment have recognized or unrecognized neurodevelopmental disorders (NDD) (4, 5), cognitive dysfunction (6), or psychiatric disorders (7). These conditions are suggested to influence obesity management negatively (2, 6). Interestingly, they seem to have a state of vitamin D insufficiency which exhibit in normal conditions neurotrophic and antioxidant effect in the central nervous system (8, 9). So far, most studies have explored NDD, cognitive dysfunction, and other psychiatric disorders separately, and little is known about the co-occurrence of NDD, cognitive dysfunction and psychiatric disorders in children undergoing obesity treatment.

NDD include a group of conditions with early developmental onset (10). The common co-occurrence of NDD, cognitive deficits and psychiatric conditions is presented as ESSENCE (Early Symptomatic Syndromes Eliciting Neurodevelopmental Clinical Examinations) (11). This concept covers conditions with early developmental onset, not only disorders but also difficulties that do not fulfill the criteria for a specific disorder; for instance, borderline intellectual functioning (BIF, *i.e.*, intellectual quotient [IQ] 70–84). Children with BIF are a vulnerable group with an increased risk of psychiatric conditions and negative effects related to educational and behavioral functioning (12). BIF is also common in NDD (11). Among NDD, attention deficit/hyperactivity disorder (ADHD) and autism spectrum disorder (ASD) have repeatedly, but separately, been shown to have high comorbidity with obesity (4, 13, 14). However, there is a lack of studies evaluating a broader range of NDD in relation to obesity.

IQ is a measure of general cognitive functioning and the most robust and reliable measure to predict global functioning and health outcomes (15, 16). Certain studies have indicated lower IQ scores in children with obesity compared with normal weight peers or norm data; however, the results are inconclusive (17–19). The different results between studies may be due to lower IQ scores in some studies being confounded by the generally lower socioeconomic status among children with obesity (17, 20, 21). Contradictory findings may also be related to differences in the assessment of IQ, and abbreviated scales are often used in studies (17). Also, to the best of our knowledge, no study has so far considered NDD when studying IQ in children with obesity, even if cognitive deficits are common in patients with NDD, regardless of their weight status (11).

Furthermore, there is an increased risk of depression or anxiety both in children with obesity (7, 22) and in children with NDD (23, 24), but few studies have investigated such psychiatric conditions in children with both obesity and NDD. However, a Swedish register-based study showed that obesity was associated with an increased risk of depressive and anxiety disorders during childhood (between 6 and 18 years of age). This risk was independent of other risk factors such as NDD, sex and socioeconomic status (22). Nevertheless, register-based research relies on recorded diagnoses, and many mental health disorders may go undetected, thereby accentuating the need for more thorough clinical studies. Early detection may be crucial in preventing the progression of maladaptive development and the risk of subsequent psychiatric disorders (25).

The aim of this study was to explore the co-occurrence of NDD, with focus on ASD, ADHD, specific learning disorders including BIF and motor disorders along with internalizing and externalizing disorders in children presenting for obesity treatment, by 1) determining the frequency of diagnosed NDD (including identified BIF) and diagnosed psychiatric disorders, 2) exploring intellectual abilities and internalizing symptoms in children with and without a diagnosed NDD, and 3) exploring associations between clinical background factors, IQ, and internalizing symptoms.

## Materials and methods

### Study sample and procedure

**Figure 1:**
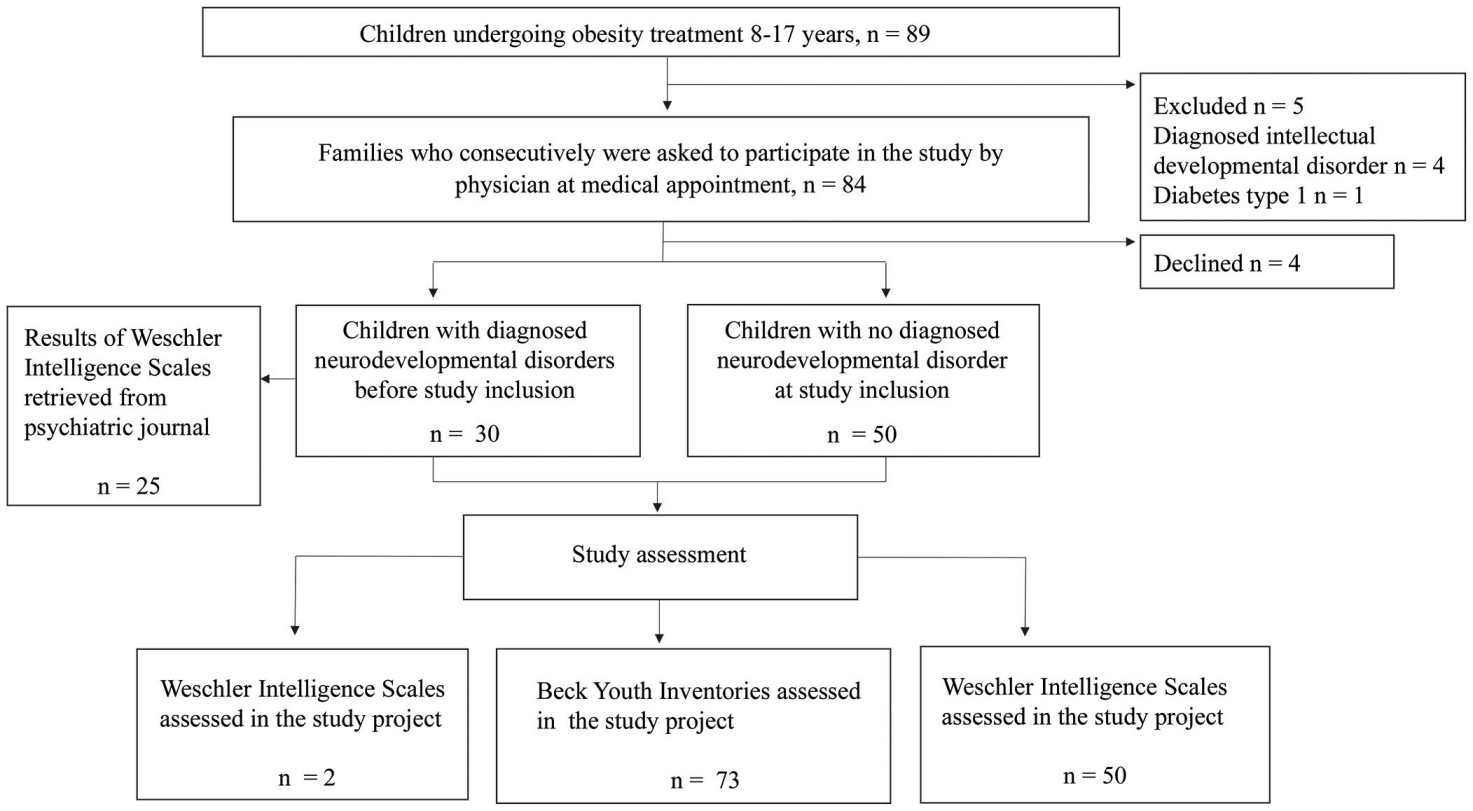
Flow chart of the recruitment procedure and time of cognitive and psychological assessment.

This observational study consecutively offered participation in the study by the pediatrician during regular medical appointments for children aged 8-17 years undergoing obesity treatment at either of two pediatric outpatient obesity clinics in the Western Region of Sweden (Kungsbacka and Varberg). The period of inclusion was 2018 and 2019. Obesity was defined as a body mass index standard deviation score (BMI SDS) of ≥ 2. Exclusion criteria were, i) conditions or medication that might influence cognitive functioning or weight (insulin treatment for type 1 diabetes mellitus [n=1], verified syndrome with hypothalamic obesity or taking anti-epileptic drugs or oral cortisone), ii) suspected/diagnosed intellectual developmental disorder (n=4), as it may be associated with genetic conditions and implies significantly impaired intellectual and adaptive functioning (10), and iii) families non-fluent in Swedish. In total, 84 children were eligible and the parents of 80 children agreed to study participation. All children with no previously diagnosed NDD underwent a standardized clinical neurodevelopmental evaluation, including an assessment of the child’s intellectual and psychological functioning. Children who screened positive for the NDD were referred to a psychiatric outpatient clinic for further evaluation for diagnosis (26). **Figure 1** displays flow chart of the recruitment procedure and time of cognitive and psychological assessment.

### Assessments

**Table 1** provides a detailed description of the assessment battery. General intellectual ability, measured as Full-Scale IQ (FSIQ), was assessed with the Wechsler Intelligence Scales, the golden standard for assessing intellectual ability. Besides FSIQ, the Wechsler Intelligence Scales measure different cognitive domains, defined as indexes (sum of scaled scores). All Wechsler Intelligence Scales used in this study included the following indexes; Verbal Comprehension, Visuo-Spatial/Perceptual Reasoning/Performance, Working Memory and Processing Speed (27).

**Table 1:** The assessment battery.

Variable	Measurement	Age criteria for measurement	Children assessed	Description
General intellectual ability**^1^**	Wechsler Intelligence Scale for Children Fifth Edition	6 – 16 years and 10 months	53	General intellectual ability measured as Full-Scale IQ. It comprises following index: Verbal Comprehension index. Visuo-Spatial index, Fluid index, Working Memory index, Processing Speed index
General intellectual ability**^2^**	Wechsler Adult Intelligence Scale Fourth Edition	16 years and 10 months – 90 years	3	General intellectual ability measured as Full-Scale IQ. It comprises following index: Verbal Comprehension index, Perceptual Reasoning index, Working Memory index, Processing Speed index
General intellectual ability**^3^**	Wechsler Intelligence Scale for Children Fourth Edition	6 – 16 years and 10 months	20	General intellectual ability measured as Full-Scale IQ. It comprises following index: Verbal Comprehension index, Perceptual Reasoning index, Working Memory index, Processing Speed index
General intellectual ability**^4^**	Wechsler Preschool and Primary Scale of Intelligence-3^rd^ Edition	2 years and 6 months – 7 years and 3 months	1	General intellectual ability measured as Full-Scale IQ. It comprises following index: Verbal IQ and Performance IQ. For children between 2:6–3:11 is also measure General Language Composite. For children between ages 4:0–7:3 Processing Speed Quotient is attainable
Anxiety and depressive symptoms	The Beck Youth Inventories	7 – 18 years	73	Self-report scales of internalizing symptoms. A total raw score is calculated for each scale and translated into percentiles using Swedish gender-specific norms

^1^Full-Scale IQ is defined by seven subtests corresponding to following indexes: Verbal Comprehension index (Similarities, Vocabulary), Visuo-Spatial index (Block Design), Fluid index (Matrix Reasoning, Figure Weights), Working Memory index (Digit Span) and Processing Speed index (Coding).

^2^Full-Scale IQ is based on the total combined performance for the indexes: Verbal Comprehension index (Similarities, Vocabulary, Information), Perceptual Reasoning index (Block Design, Matrix Reasoning, Visual Puzzles), Working Memory index (Digit Span, Arithmetic), and Processing Speed index (Coding, Symbol Search).

^3^Full-Scale IQ is composed of 10 core subtests corresponding to following indexes: Verbal Comprehension index (Vocabulary, Similarities, Comprehension); Perceptual Reasoning index (Block Design, Picture Concepts, Matrix Reasoning); Working Memory index (Digit Span, Letter-Number Sequencing); Processing Speed index (Coding, Symbol Search).

^4^Full-Scale IQ is based on Verbal IQ (Information, Vocabulary, Word Reasoning) and Performance IQ (Block Design, Matrix Reasoning, Picture Concepts) and Coding.

A clinical psychologist (MDB) assessed all children with no diagnosed NDD at study start; 47 children were assessed with the Wechsler Intelligence Scale for Children-5^th^ Edition (WISC-V) (28) and the remaining three children with Wechsler Adult Intelligence Scale-4^th^ Edition (WAIS-IV) (29). For children previously diagnosed with an NDD, it was assumed that they had already undergone a cognitive assessment as it is a standard part of a neurodevelopmental evaluation. Hence, they were not assessed again, instead we extracted the cognitive results from their psychiatric records. However, at follow-up it was discovered that four children had not undergone a cognitive assessment when diagnosed with an NDD. Two of them underwent assessment with the WISC-V (28) by the psychologist (MDB), whereas two patients (> 18 years at follow-up) were no longer patients at the obesity clinic and were therefore omitted from the cognitive part of the study. Among the other children previously diagnosed with an NDD, the following versions of the Wechsler Intelligence Scales had been used: one child had been assessed with the Wechsler Preschool and Primary Scale of Intelligence-3^rd^ Edition (30), while 20 children had been assessed with the Wechsler Intelligence Scale for Children-4^th^ Edition (WISC-IV) (31) and additional four participants with the WISC-V (28). The WISC-V has been examined in relation to previous editions of the instrument and has shown good correlation (27).

The Wechsler Intelligence Scales assume normal distribution, and raw scores are converted into scaled scores using age-matched norm data. The mean (standard deviation [SD]) of the FSIQ and indexes correspond to 100 (15) and can be qualitatively categorized: extremely low (-69), very low (70-84), low average (85-92), average (93-107), high average (108-115), very high (116-130), and extremely high (131-) (27).

None of the participants were taking psychostimulants at the time of the cognitive assessment. The timepoint of the cognitive assessment (age of the child at cognitive testing) was categorized as the starting point of the diagnostic evaluation process. Per definition this categorization excludes those two previously diagnosed NDD children who were assessed at our study follow-up.

The Beck Youth Inventories (BYI) (age 7-18 years) (32) consist of five reliable (mean *Cronbach’s α* ≥.89) self-report scales that can be used separately or in combination. The inventories for anxiety and depression (internalizing symptoms) were used in this study. A total raw score is calculated for each scale and translated into percentiles using Swedish gender-specific norms. A higher percentile reflects more symptoms. The 90^th^ percentile corresponds to highly elevated symptoms and can be used as a clinical cut-off. All children filled out BYI during the time of the study project; for children screened in the study at the time of cognitive testing, and for the participants previously diagnosed with an NDD, either at their medical appointment or at home.

### Retrieval and classification of neurodevelopmental and psychiatric diagnoses

In the present study NDD included ASD, ADHD, motor disorders as well as specific learning disorders (e.g., dyslexia, dyscalculia) (10). In this study specific learning disorders were grouped together with, by psychiatric journal, identified BIF and they were categorized as “any learning disabilities”. Further, ADHD was stratified by its different subtypes: predominantly inattentive, predominantly hyperactive/impulsive, and combined. This study also examines the frequency of depressive disorders, anxiety disorders, obsessive-compulsive and related disorders, trauma-compulsive and stressor-related disorders as well as disruptive, impulse-control, and conduct disorders (10). In the current study these disorders are denoted as psychiatric disorders. Psychiatric disorders were categorized as internalizing disorders (the propensity to express distress inwards) or externalizing disorders (the propensity to express distress outwards) (33). The first author (MDB) extracted the diagnoses and identified BIF from psychiatric records two years after the data collection finished in May 2021, to ensure that the psychiatric unit had received sufficient time to assess the children. For children already diagnosed with an NDD, the diagnoses were also confirmed through the records at the time of consent.

### Patient characteristics

Height and weight were measured by a nurse at the clinic with standardized methods and BMI SDS was calculated (34). Sex, age, and BMI SDS were then obtained from medical records from the date the child started the obesity treatment at the clinic, at time of cognitive testing, at inclusion and at follow-up.

The highest educational level of parents in the family was used to defined socioeconomic status. Parental educational level was retrieved from Statistics Sweden corresponding to the year 2018 and is reported in accordance with SUN 2020 (35). In this study, lower educational level involved pre-primary education, primary and lower secondary education less than 9 years and primary and lower secondary education 9 (10) years, whereas advanced educational level was defined as upper secondary education, post-secondary education less than 2 years, post-secondary education 2 years or longer as well as postgraduate education.

### Statistics

Descriptive data are presented as means (SD), medians (inter-quartile range [IQR]), or frequencies (N; %). For continuous variables, comparisons between groups were analyzed using the independent sample t-test or ANOVA (if normally distributed), Kruskal-Wallis or Mann-Whitney U (if non-normally distributed), and Fischer’s exact test (low frequencies). Shapiro-Wilks (p ≤ 0.05) and histograms were used to identify non-normal distributions. The Pearson correlation was used for the correlation analysis. The degree of correlation was defined as follows: coefficient value r; small ≤ 0.29; medium 0.3- 0.49; and strong 0.5 – 1. For parametric tests, a p value ≤ 0.05 was considered statistically significant. For non-parametric tests, a p value ≤ 0.10 defined significance due to small sample sizes and great variations within the subgroup BYI scores (36). All statistical analyses were made using IBM SPSS Statistics version 27 (IBM SPSS Statistics, Chicago, IL).

### Ethics

The Regional Ethical Review Board in Gothenburg (reg. no. 409-14) approved the study. Written informed consent was obtained from parents and assent from patients if their age was ≥ 12 years. The study was carried out in line with the Declaration of Helsinki.

## Results

**Table 2** presents the patient characteristics. One of the screened patients turned out to have a suspected intellectual developmental disorder and was omitted from analysis. We found 30/79 (38%) participants already diagnosed with an NDD when they were included in this study and are henceforth denoted the “pre-diagnosed NDD group”. Additionally, 17/50 (34%) children undergoing screening in this study were subsequently diagnosed with an NDD after referral to the psychiatry unit (26) and are henceforth denoted the “study-diagnosed NDD group”. The remaining 32 participants are denoted the “non-NDD group”. The pre-diagnosed NDD group was significantly older at the time of completing the BYI compared with the study-diagnosed NDD group (*P = .*02). The delta mean BMI SDS between obesity treatment start and follow-up for the study-diagnosed NDD group was -0.5 SDS. Additionally, a greater number of parents in the non-diagnosed NDD group had advanced educational level compared to pre-diagnosed NDD group (*P = .*07). The corresponding analysis between non-diagnosed NDD group and study-diagnosed group did not reach a significant level (*P = .*20).

**Table 2:** Characteristics of children with obesity seeking treatment included in the study, broken down by group.

	Pre-diagnosed NDD group N = 30	Study-diagnosed NDD group N = 17	Non-NDD group N = 32	P value
Sex distribution				
Boys N (%)	21 (70)	8 (47)	14 (44)	.09
Age at				
Treatment start (t1) Study start Cognitive testing Beck Youth Inventory (t2) Follow-up (t3)	11.3 (2.37) 13.7 (2.59) 10.5 (2.41)^1^ 14.6 (2.65)^3^ 16.0 (2.72)	9.7 (2.86) 11.6 (3.30) 12.0 (3.29) 12.0 (3.29) 13.7 (2.74)^5^	11.0 (2.57) 12.6 (3.11) 13.1 (3.00) 13.1 (3.00) 14.9 (3.03)^6^	.13 .06 <.01^2^ .02^4^ .04^7^
BMI SDS^8^ at				
Treatment start (t1) Study start Cognitive testing Beck Youth Inventory (t2) Follow-up (t3)	2.8 (0.49) 2.8 (0.56) 2.6 (0.80) 2.6 (0.89)^3^ 2.9 (1.12)	2.8 (0.46) 2.8 (0.63) 3.0 (0.57) 3.0 (0.57) 2.4 (0.75)^5^	2.9 (0.57) 2.9 (0.50) 2.9 (0.52) 2.9 (0.52) 2.8 (1.14)^6^	.71 .77 .08 .14 .23
Delta data				
Delta time (years) t1-t2 Delta BMI SDS t1-t2 Delta time (years) t1-t3 Delta BMI SDS t1-t3	3.9 (2.17)^3^ -0.2 (0.76)^3^ 4.7 (2.45) 0.1 (0.99)	2.2 (2.17) 0.17 (0.62) 4.3 (1.95)^5^ -0.5 (0.73)^5^	2.1 (1.79) 0.0 (0.55) 3.7 (2.02)^6^ 0.0 (0.94)^6^	<.01^9^ .16 .20 .14
Parental educational level N (%)				
Lower educational level Advanced educational level	15 (50) 15 (50)	8 (47) 9 (53)	8 (25) 24 (75)	.10^10^

Data are expressed as means (SD) or N (%). P value calculated with ANOVA or with Chi-square test. NDD, neurodevelopmental disorders.

^1^n = 27. ^2^P <.01 only between non-NDD group and pre-diagnosed NDD. ^3^n = 24. ^4^ *P* = .02 only between study-diagnosed NDD group and pre-diagnosed NDD group. ^5^n = 16, ^6^n = 30, ^7^ *P* = .03 only between study-diagnosed NDD group and pre-diagnosed NDD group. ^8^Body mass index standard deviation score. ^9^ *P* = .03 between study-diagnosed NDD group and pre-diagnosed NDD group, *P* <.01 between non-NDD group and pre-diagnosed NDD group. ^10^ *P* = 1.00 between pre-diagnosed NDD group and study-diagnosed NDD group, *P* = .07 between pre-diagnosed NDD group and non-NDD group, *P* = .20 between study diagnosed NDD group and non-NDD group.

**Table 3** displays the frequency and distribution of the diagnoses in the subgroups, and the merged pre-diagnosed and study-diagnosed NDD groups (denoted “NDD total group”) and the total study sample. At follow-up, a total of 47 patients were diagnosed with an NDD and 45 of these had ADHD (96%). Fourteen participants had ASD (30%) and twelve of them also had ADHD. Eighteen of 47 (38%) patients had more than one identified comorbidity; six children (13%) had ADHD with any learning disability of which one also had with tic disorder, 11 children (23%) had ADHD with ASD including one who had additionally been diagnosed developmental coordination disorder and one child (2%) had ASD and ADHD with any learning disability. In addition to ADHD, internalizing and externalizing disorders were diagnosed in 21 of 79 participants (27%). Depressive disorder was the most common. All but one child in the pre-diagnosed group had been diagnosed with internalizing and/or externalizing disorder before study inclusion, while children in the study- diagnosed NDD group and non-diagnosed NDD group were diagnosed with these psychiatric disorders during study follow-up.

**Table 3:** Distribution of neurodevelopmental disorders (NDD) including any learning disability ^1^ and psychiatric disorders in children presenting for obesity treatment, broken down by group.

NDD diagnoses	Pre-diagnosed NDD group N = 30	Study-diagnosed NDD group N = 17	Non-NDD group N = 32	NDD total group N = 47	Total study sample N = 79
Attention deficit/hyperactivity disorder (ADHD), totally Autism spectrum disorder (ASD), totally ADHD without ASD, totally ADHD with ASD, totally	28 (93) 13 (43) 17 (57) 11 (37)	17 (100) 1 (6) 16 (94) 1(6)	N/A	45 (96) 14 (30) 33 (70) 12 (26)	45 (57) 14 (18) 33 (42) 12 (15)
Specific subtypes of ADHD					
ADHD combined only ADHD combined/any learning disability ASD/ADHD combined ASD/ADHD combined/any learning disability ADHD inattentive only ADHD inattentive/any learning disability ASD/ADHD inattentive	7 (23) 5 (17)^2^ 7 (23)^3^ 1 (3)^4^ 5 (17) 0 (0) 3 (10)	6 (35) 0 (0) 0 (0) 0 (0) 9 (53) 1 (6)^5^ 1 (6)		13 (28) 5 (11)^2^ 7 (15)^3^ 1 (2)^4^ 14 (30) 1 (2)^5^ 4 (9)	13 (16) 5 (6) 7 (9) 1 (1) 14 (18) 1 (1) 4 (5)
Psychiatric disorders^6^					
Internalizing disorders, totally Externalizing disorders, totally	11 (37) 2 (7)	3 (18) 1 (6)	4 (13) 0 (0)	14 (30) 3 (6)	18 (23) 3 (4)
Specific subtypes of psychiatric disorders					
**Internalized disorders** Depressive disorder only Depression/anxiety disorders Anxiety disorder only Obsessive-compulsive disorder	9 (30) 1 (3) 0 (0) 1 (3)	0 (0) 2 (12) 1(6) 0 (0)	1 (3) 2 (6) 1 (3) 0 (0)	9 (19) 3 (6) 1 (2) 1 (2)	10 (13) 5 (6) 2 (3) 1 (1)
**Externalized disorders** Oppositional defiant disorder	2 (7)	1(6)	0 (0)	3 (6)	3 (4)

The frequencies are presented as N (%). NDD, neurodevelopmental disorders.

^1^Any learning disability involves dyslexia, dyscalculia or identified borderline intellectual functioning. ^2^n=5 had any learning disability, of which one also had with Tic disorder. ^3^n=1 was additionally diagnosed developmental coordination disorder. ^4^Any learning disability n=1. ^5^Any learning disability n=1. ^6^All but one child in the pre-diagnosed NDD group were diagnosed with internalized or externalized disorders before study inclusion. For children in the study-diagnosed NDD group and non-diagnosed NDD group, children were diagnosed during study follow-up.

As expected, pre-diagnosed NDD children were significantly younger, 10.70 (3.10) years, at the time of receiving their first NDD diagnosis, compared with study-diagnosed NDD children, 12.78 [(3.11), *P* = .03] years. In the pre-diagnosed group, 73% were boys (22/30), compared with 47% (8/17) in the study-diagnosed group. The sex distribution was not significantly different between the groups (*P* = .12). **Table 4** presents number of NDD-related comorbidities in the pre-diagnosed NDD group compared with the study-diagnosed NDD group. Additionally, significantly more pre-diagnosed NDD boys were diagnosed with ASD compared with the study-diagnosed NDD boys (*P = .*01). A significantly larger number of study-diagnosed NDD girls were diagnosed with ADHD of the inattentive subtype, compared with pre-diagnosed NDD girls (*P* = .02).

**Table 4:** Comparison of neurodevelopmental-related comorbidities between children diagnosed with neurodevelopmental disorders before study start (pre-diagnosed NDD group) and children diagnosed with neurodevelopmental disorders by study screening (study-diagnosed NDD group).

	Pre-diagnosed NDD group N = 30	Study-diagnosed NDD group N = 17	P value
**Number of neurodevelopmental disorders** N One neurodevelopmental disorder**/**Two or more neurodevelopmental disorders	14/16	15/2	.01
**Distribution of specific neurodevelopmental disorders** N (%) ADHD, totally Combined ADHD subtype Inattentive ADHD subtype Autism spectrum disorder^1^	28 (93) 20 (71) 8 (27) 13 (43)	17 (100) 6 (35) 11 (65) 1 (6)	.53 .04 .01 .01

Data are presented as N (%). P value calculated with Fisher exact test. NDD, neurodevelopmental disorders.

^1^Including children with any additional neurodevelopmental comorbidity (e.g., ADHD), identified dyslexia or borderline intellectual functioning (any learning disability) but excluding intellectual disability.

The FSIQ results are presented in **Table 5** and the additional indexes are shown in **Supplementary Table S1**. Qualitatively categorized FSIQ was accessible for 76 out of 79 participants, showing that 21 participants (28%) performed within the very low range and one child in the very high. In addition, the NDD total group scored significantly lower on the FSIQ, 89.1 (10.0), compared with the non-NDD group, 95.7 (12.1), (*P* =.01). The pre-diagnosed NDD group performed worse on the Working Memory index (*P* = .01) and Processing Speed index (*P* = .02) relative to the non-NDD group, while the study-diagnosed NDD group achieved lower scores on the Working Memory index compared with the non-NDD group (*P* = .03). No significant differences were detected between the study-diagnosed and the pre-diagnosed NDD group.

**Table 5:** Full-Scale IQ of Wechsler Intelligence Scales in children seeking obesity treatment.

Wechsler Intelligence Scales	Pre-diagnosed NDD group N = 27	Study-diagnosed NDD group N = 17	Non-NDD group N = 32	P value
	Mean (SD)	Mean (SD)	Mean (SD)	
Full-Scale IQ	89.3 (10.3)^1^	88.8 (10.0)	95.7 (12.1)	.05^2^
Qualitative description	N (%)	N (%)	N (%)	Total N (%)
Reference: *(Normal distribution %)*				
Extremely Low *(2.2)*	0 (0)	0 (0)	0 (0)	0 (0)
Very Low *(6.7)*	8 (29.6)	6 (35.3)	7 (21.9)	21 (27.6)
Low Average *(16.1)*	9 (33.3)	7 (41.2)	8 (25.0)	24 (31.6)
Average *(50)*	9 (33.3)	3 (17.6)	11 (34.4)	23 (30.2)
High Average *(16.1)*	1 (3.7)	1 (5.9)	5 (15.6)	7 (9.2)
Very High (6.7)	0 (0)	0 (0)	1 (3.1)	1 (1.3)
Extremely High *(2.2)*	0 (0)	0 (0)	0 (0)	0 (0)

P-value was accounted with ANNOVA.

^1^n=22. ^2^ No significant differences were seen in post-hoc tests.

At time of study assessment, 24 of 73 (33%) scored > the 90^th^ percentile on the anxiety inventory on the BYI. The corresponding number for the depression inventory was also 33%. Totally, 33 (45%) of the participants who completed the BYI (n=73) reported at least one of the anxiety or depression inventories > the 90^th^ percentile. Of the 33 participants reporting > the 90^th^ percentile, 15 (45%) reported both highly elevated symptoms of depression and anxiety, while 18 (55%) reported one of the inventories > the 90^th^ percentile (either depression or anxiety). No significant sex differences were found for symptoms of depression (*P = .*50) or anxiety (*P = .*31).

**Table 6** presents data for internalizing symptoms, compared between subgroups. The pre-diagnosed NDD group scored higher for depressive symptoms than the study-diagnosed NDD group, but it did not reach a statistical significant level. However, ten participants in the pre-diagnosed NDD group were already on treatment for depressive disorder. Three of them did not complete the BYI due to lack of energy related to depression, whereas four of the ten scored < the 90^th^ percentile.

**Table 6:** Internalizing symptom scores assessed with the Beck Youth Inventory.

Beck Youth Inventory (Percentiles)	Pre-diagnosed NDD group N = 24	Study-diagnosed NDD group N = 17		Non-NDD group N = 32	
	Median (IQR)	Median (IQR)		Median (IQR)	
Anxiety inventory	82.70 (35.00)	63.60 (62.20)		79.35 (42.20)	
Depression inventory	82.80 (28.80)	45.10 (68.90)		72.20 (61.60)	
Anxiety percentile	Test statistics	Sth. Error	Sth. Test Statistics	P value	P value adj.
Pre-diagnosed NDD group Study-diagnosed NDD group	-9.15	6.72	-1.36	.17	.52
Non-NDD group Pre-diagnosed NDD group	.84	5.72	.14	.88	1.00
Non-NDD group Study-diagnosed NDD group	-8.30	6.36	-1.31	.19	.58
Depression percentile					
Pre-diagnosed NDD group Study-diagnosed NDD group	-12.57	6.72	-1.87	.06	.19
Non-NDD group Pre-diagnosed NDD group	4.68	5.73	.82	.41	1.00
Non-NDD group Study-diagnosed NDD group	-7.89	6.36	-1.24	.22	.65

Results are compared with regard to undiagnosed or diagnosed neurodevelopmental disorder (NDD), stratified by when receiving the NDD diagnosis.

Associations between clinical background factors, FSIQ and internalized symptoms for all subgroups and the total study sample are presented in **Table 7**. Higher BMI SDS at cognitive testing was significantly associated with lower FSIQ scores among non-NDD children (*P = .*02). This association was not seen in the other groups. Only in the total sample, children of parents with lower educational level scored significantly poorly on FSIQ compared to children of parents with advanced educational level (*P = .*04). Also, age at BYI inventory and anxiety symptoms (r = .49, *P = .*04) and depressive symptoms (r = .78, *P* <.001) were positively correlated in the study-diagnosed NDD group. For the NDD total group, older age at cognitive testing (starting point of diagnostic evaluation process) was significantly associated with higher FSIQ scores (r = .35, *P = .*03) and, similarly, a higher FSIQ was significantly associated with anxiety symptoms (r = .40, *P = .*02). The correlation between older age at the starting point of the diagnostic evaluation process and anxiety symptoms did not reach significance (r = .30, *P = .*07). The corresponding correlation for depressive symptoms was significant (r = .35, *P = .*03).

**Table 7:** Association between clinical background factors, Full-Scale IQ (Weschler Intelligence Scales), depressive percentile and anxiety percentile (Beck Youth Inventory; BYI), broken down by group: children diagnosed with neurodevelopmental disorder before study start (pre-diagnosed NDD group)^1^, children diagnosed with neurodevelopmental disorder by study screening (study-diagnosed NDD group) and children not diagnosed with any neurodevelopmental disorder (non-diagnosed NDD group), children with diagnosed neurodevelopmental disorder total group (NDD total group)^1^ as well as total study sample.

	Pre-diagnosed NDD group N = 24		Study-diagnosed NDD group N = 17		Non-NDD group N = 32		NDD total group N = 41		Total study sample N = 73	
Associations with Full-Scale IQ										
	Median (IQR)	P value	Median (IQR)	P value	Median (IQR)	P value	Median (IQR)	P value	Median (IQR)	P value
Parental educational level Lower educational level Advanced educational level	88 (15) 88 (17)	1.00	84 (10) 92 (14)	.15	87 (16) 98 (18)	.22	86 (13) 89 (14)	.43	86 (13) 92 (16)	.04

	R	P value	R	P value	R	P value	R	P value	R	P value
Age at cognitive testing	.35	.13^2^	.38	.14	.01	.94	.35	.03^6^	.25	.04^9^
BMI SDS**^3^** at cognitive testing	.05	.83^4^	-.02	.94	-.42	.02	.02	.91^7^	-.21	.09^10^
Associations with Depression percentile										
Age at cognitive testing	.09	.71^2^	.78	<.001	.32	.08	.35	.04^6^	.32	<.01^9^
Full-scale IQ	-.14	.59^5^	.28	.28	.24	.19	.11	.54^8^	.17	.17^11^
Age at BYI	.27	.20	.78	<.001	.32	.08	.46	<.01	.39	<.001
BMI SDS**^3^** at BYI	.17	.44	.14	.59	.19	.29	.03	.84	.09	.48
Associations with Anxiety percentile										
Age at cognitive testing	.23	.33^2^	.49	.04	-.07	.72	.30	.07^6^	.19	.12^9^
Full-scale IQ	.43	.08^5^	.44	.08	.29	.11	.40	.02^8^	.34	<.01^11^
Age at BYI	.02	.91	.49	.04	-.07	.72	.33	.03	.18	.14
BMI SDS**^3^** at BYI	.34	.10	-.02	.94	-.10	.60	.14	.39	.08	.50

^1^The two pre-diagnosed NDD children who were cognitively assessed at follow-up were excluded from analysis. Accordingly, age at cognitive testing was regarded as the time of starting the diagnostic evaluation process. ^2^n = 20 ^3^Body mass index standard deviation score ^4^n = 22 ^5^n = 17 ^6^n = 37 ^7^n = 39 ^8^n = 34 ^9^n= 69 ^10^n = 71 ^11^n = 66.

## Discussion

This study examined a wide spectrum of neurodevelopmental and psychiatric comorbidities in 80 consecutively recruited children from two pediatric obesity outpatient clinics in Sweden. At least one NDD was found in 59% of the children with obesity, and all but two with NDD had an ADHD diagnosis. Almost 20% of the children with an NDD had ASD. The proportion of children in obesity treatment with ADHD varies between studies, and frequencies between 18% (5) and 58% (37) have been reported. Among previous publications in children with ASD, 17% also had obesity (13).

The clinical presentations in this group of children with obesity fit well into the concept of ESSENCE (11). This is one of the novel findings in the present study. Nearly two fifths (38%) of the children with obesity diagnosed with a NDD had more than one identified NDD defined comorbidity. Another marker of ESSENCE is cognitive impairments and in our study children with NDD showed greater cognitive deviances compared to non-NDD diagnosed children, especially the pre-diagnosed NDD group. Also 33% of the pre-diagnosed children turned out to have depression at follow-up.

Children diagnosed with an NDD before the study were characterized by two or more early-onset developmental problems and disorders such as ASD and/or a combined ADHD subtype. Additionally, ASD was more prevalent in pre-diagnosed boys compared with study-diagnosed NDD boys. Both co-occurring NDDs, which may lead to more severe problems with adaptive functioning (38), and hyperactivity and/or disruptive behavior (23, 24, 39), may enable early detection. A larger proportion of study-diagnosed girls had inattentive ADHD compared with the pre-diagnosed NDD girls. Compared with combined or hyperactive/impulsive ADHD, inattentive ADHD is more common in girls than boys and may be less noticeable to others. This may explain why ADHD in girls is easily overlooked and often identified at an older age (24).

Our study indicates that NDD in children presenting for obesity treatment could be at risk of a delayed NDD diagnosis (40–42) possibly affecting obesity treatment outcome. The participants mean age when receiving the first NDD diagnosis occurred during middle school age, for both the pre-diagnosed and study-diagnosed NDD group (10.7 and 12.8, respectively). The recommended treatment for ADHD is to apply a multimodal approach including medication and psychopedagogical interventions (43, 44). Early detection, prior to starting school, may lead to earlier treatment and better chance of preventing school failure and overweight (17, 24, 45).

In the present study, the results of the depressive diagnosis (see **Table 3**) and the depressive symptoms (see **Table 6**) are in line with each other. Children diagnosed with an NDD, especially pre-diagnosed NDD children, developed more often depressive symptoms. This trend is probably also driven by age, as the pre-diagnosed NDD children were older compared with the study-diagnosed children. Additionally, only in the NDD group diagnosed in the study, a positive correlation was observed between age at BYI and internalizing symptoms. The influence of age does not seem implausible (46). The prepubertal age has been found to be a special vulnerable period for children with NDD and has been linked to the increase of school and psychosocial demands that comes with an older age (47, 48). Also, for NDD subgroups, a delayed diagnosis may increase the burden of emotional problems due to lack of early adequate support (23, 24, 45). One other explanation might be a surveillance bias, as the pre-diagnosed NDD children were already followed up more closely than the other participants at the psychiatry unit. It is also possible that the higher rate of behavioral comorbidities in addition to obesity, seen in the pre-diagnosed NDD group, could add to the strain of coping with situations in everyday life such as school (38). The severity of the NDD could possibly have a negative effect on emotional development (45, 47) and, thereby, weight management, as these factors are known to have a reciprocal relationship (7). Environmental factors are other examples, such as the steroid hormone vitamin D, which modulates gene regulation decreasing the risk of developing ASD and depression (8, 49, 50). As previously mentioned, NDD as well psychiatric disorders, are found to have vitamin D deficiency (8, 9). This is also true for children with obesity (51), but the pathophysiological mechanisms are not fully understood.

In addition to psychiatric outcomes, we found for the study-diagnosed NDD group the mean delta BMI SDS from obesity treatment start to follow up to be -0.5 SDS, which is considered clinically relevant (52). At follow-up, these children had been diagnosed at the psychiatry unit and support had been initiated. The exact reason for this difference in treatment success is not known, but it could be speculated that an ADHD diagnosis may contribute to tailor the support in obesity treatment, making it more effective (4). Also, psychostimulant treatment for ADHD has been proven to yield significant weight loss (53).

Lower IQ scores in childhood have been suggested to precede the persistence of obesity in adulthood, but this relationship is plausibly mediated by educational progress (17). The mean level FSIQ for all groups fell into the average range. Still, a substantial number of the participants performed worse than expected; 28% of the total sample scored in the BIF range (12). The corresponding expected proportion in a population sample is 6.7% (27). Being identified with BIF is important for the treatment of psychiatric disorders and makes it possible to enhance adapted school situations (54).

We also found the NDD total group to score significantly lower on FSIQ than the non-NDD group. Children with ADHD as a group (regardless of their weight status) has been found to exhibit mild intellectual difficulties compared with healthy controls (55). Interestingly, we did not find any significant relationship between BMI SDS and FSIQ in the NDD groups, but in the non-NDD group, a higher BMI SDS was related to a lower FSIQ score. This finding needs further studies to understand the mechanisms behind. Additionally, children of parents with lower educational level performed more poorly on FSIQ compared to children of parents with advanced educational level. However, this was only significant when not splitting into subgroups. This suggest that it may be an underlying factor in each group but may be biased by the small sample sizes in this study. Studies with larges sample sizes are needed where cognitive function should be investigated from a more multifaceted perspective and thereby evaluating relationships between variables.

Somewhat surprising was that a higher FSIQ score (although mainly within the lower and average range) correlated with anxiety symptoms for the NDD total group. Even though intellectual difficulties have been found to place children at risk of internalizing symptoms (56, 57), a relative higher IQ may partly explain why an NDD more easily remains undetected in some children until school age. It is believed that it could compensate for and/or overshadow (“hide”) other behavioral difficulties and be one of several reasons for delaying the start of the NDD diagnosis procedure (24). A delayed diagnosis is also believed to be one of the reasons why these children develop aggregated stress and emotional problems with increasing age, as the compensatory strategies may put further strain on the child (23, 24, 58). For the NDD total group we observed associations between FSIQ, age at the start of the diagnostic evaluation process and anxiety. This may indicate an indirect effect of age when starting the NDD diagnostic procedure of anxiety symptoms, which is mediated by the FSIQ. This underlying relationship might exist in each of the two NDD subgroups, but due to insufficient sample sizes it may only emerge when the NDD groups are combined. These correlation patterns emerged despite screening one of the groups, and that may have biased the result.

Treatment adherence and school absence are known problems in childhood obesity (3, 59). Successful modification of lifestyle habits and school attendance may be hampered by interactions of emotional, cognitive, and behavioral problems (7, 60). In addition to our results of NDD, we found more than two fifths of the participants (45%) at time of study assessment to self-report anxiety and/or depression above the clinical threshold compared to norms. At follow-up, 22% were diagnosed with depression and/or anxiety disorder/s. It is known that ADHD and a depression symptomatology may overlap (restlessness, irritability, and difficulty concentrating) (61), why our findings suggest that obesity units would benefit from psychiatric competence, allowing for a broad screening of neurodevelopmental and psychiatric conditions to provide adequate support. Early recognition offers the greatest opportunities for adequate adaptations and support in the hope of avoiding worsening of the problems observed (25).

### Strength and limitations

To our knowledge, this is the first study to explore and investigate NDD, psychiatric conditions and cognitive ability in children presenting for obesity treatment. Main strengths include the thorough assessment and the high participation rate (95%). Also, the NDD diagnoses were evaluated at the psychiatry unit that was not involved in this study.

A limitation is the lack of a control group, but the instruments that were used allowed comparisons with normative population data. Another limitation is the exact collection of treatment protocols and data on adherence to these. Also, 80 participants should be regarded as a small sample and the study might have been under-powered to detect important differences between the groups. Cognitive functions and psychiatric conditions were assessed, but we lack information about adaptive functioning. Furthermore, the following should be considered when interpreting the results 1) numerical scores of the Wechsler Intelligence Scales were less accessible for the pre-diagnosed NDD group 2) the study did not include all type of neurodevelopmental disorders such as intellectual developmental disorder, which may affect the results found for cognitive data 3) multiple statistical comparisons were made without correction increasing the risk for false-positive results.

## Conclusion

The overlap between NDD, cognitive deficits and psychiatric conditions is an important observation for clinical practice. The present study accentuates regular screening of NDD and psychiatric conditions as an important part of obesity best practice and may benefit the field of personalized childhood obesity treatment.

## Data Availability

The raw data supporting the conclusions of this article will be made available by the authors, without undue reservation.
