# Supplementary material for: Neurodevelopmental disorders in children seeking obesity treatment- associations with intellectual ability and psychiatric conditions
Source: Front Psychiatry. 2024 Aug 19;15:1332598. doi: 10.3389/fpsyt.2024.1332598 (PMC11366696; doi:10.3389/fpsyt.2024.1332598)
Supplement: Supplementary file 1 [file Table1.docx]

**Table S1** Results on the Weschler Intelligence Scales indexes with regard to undiagnosed or diagnosed neurodevelopmental

disorder (NDD), stratified by timepoint when receiving the NDD diagnosis. Comparisons between subgroups are calculated with

ANNOVA. The P value for the post-hoc test is presented with numbers

|  | Pre-diagnosed NDD group  N = 25 | Study-diagnosed NDD group  N =17 | Non-NDD group  N =32 | | P value |
| --- | --- | --- | --- | --- | --- |
| Wechsler Intelligence Scale indexes | **M (SD)** | **M (SD)** | **M (SD)** |  | |
| Verbal Comprehension | 90.5 (11.46) | 93.7 (10.13) | 98.5 (12.65) | .04^1^ | |
| Visual Spatial/Perceptual Reasoning | 102.5 (12.44) | 93.6 (7.95) | 99.1 (13.81) | .08 | |
| Working Memory | 83.7 (12.29)^2^ | 84.8 (9.58) | 94.1 (12.29) | < .001^3^ | |
| Processing Speed | 88.3 (12.37)^2^ | 93.0 (10.98) | 97.7 (13.69) | .03^4^ | |
| *1. P* = .03 between non-NDD group and pre-diagnosed NDD group. *2.* N = 24. *3. P* = .03 between non-NDD group and study-diagnosed NDD group; *P* = .01 between non-NDD group and pre-diagnosed NDD group. *4. P* = .02 between non-NDD group and pre-diagnosed NDD group. | | | | | |
